# Vertical stratification of bacteria and the chemical compounds in crude oil-contaminated soil layers of the semi-deserted Dzungharian Basin

**DOI:** 10.1371/journal.pone.0203919

**Published:** 2018-09-25

**Authors:** Jiang-Ke Yang, Jian-Fang Liang, Lu-Mei Xiao, Yang Yang, Qun-Fang Chao

**Affiliations:** 1 College of Biology and Pharmaceutical Engineering, Wuhan Polytechnic University, Wuhan, China; 2 College of Life Science and Technology, Xinjiang University, Urumqi, Xinjiang, China; CAS, CHINA

## Abstract

The largely semi-deserted and deserted Dzungharian Basin sites in the northwest of China geologically represent an extension of the Paleozoic Kazakhstan Block and were once part of an independent continent. For reasons of overdevelopment and unreasonable operations during the process of exploitation and transportation, oil pollutants that were discharged into the soil environment caused serious pollution in this weak ecosystem. To explore the bacterial community composition in detail and their possible origination and potential during the natural attenuation of petroleum contaminants in this type of ecologic niche, GC-MS and high-throughput sequencing techniques were used to resolve the organic compounds and bacterial communities in vertical soil layers. The degradation of petroleum contaminants in semi-deserted and deserted soils mainly occurred in the layer at a depth of 45–55 cm. During this process, aromatic and heterocyclic compounds were significantly enriched in soils. The bacterial communities in this basin exhibited a distinct vertical stratification from the surface layer down to the bottom soil layer. Considering the interaction between the community composition and the geochemical properties, we speculate that the degradation of petroleum contaminants in this semi-deserted and deserted soil might represent a microorganism-mediated process and mainly occur in the deeper soil layer.

## Introduction

The Dzungharian Basin is located in the northwest of China. It is largely a semi-deserted and deserted basin surrounded by high mountains such as Mount Imeon in the south and Altai in the north. Geologically, the Dzungharian Basin is an extension of the Paleozoic Kazakhstan Block and was once part of an independent continent before the Altai Mountain formed in the late Paleozoic [1, 2]. The Dzungarian Basin is a structural basin with large estimated crude oil reserves. Petroleum mining from the basin was started in the 1950s. For reasons of overdevelopment and unreasonable operations during the process of exploitation and transportation, the oil pollutants discharged into the soils have caused serious pollution in this weak ecosystem.

Petroleum-derived contaminants constitute one of the most prevalent sources of environmental contamination in the world. Crude oil and petroleum products can all permanently contaminate the soil and waterbody, leach into the underlying groundwater system, deteriorate these ecosystems and threaten the health of organisms living in them [3, 4]. Bioremediation has emerged in recent years as a prospective measure to counteract these pollutants. During the bioremediation process, engineering measures such as selectively inoculating the bacteria agency and feeding bacteria with nutrients were generally used to accelerate the decomposition and demineralization of organic matter by improving the catabolism of microorganisms [5–7]. However, in most of the cases, especially those in deserted areas, the degradation of contaminants was still a natural attenuation process, depending on the hydrocarbon-utilizing microorganisms inhabiting the local ecosystems.

The indigenous microbial communities inhabiting local environments are valuable resources for inoculum selection for bioremediation. Resolution of the microbial communities in the contaminated sites has enriched our understanding of the magnitude of the diversity, richness, distribution and dynamics of the microbial communities in polluted sites, and it has also facilitated the selective inoculation of bacterial agents into the polluted sites to significantly improve the efficiency of bioremediation. At present, the microbial community compositions in ecological environments such as crude oil-contaminated soils [7, 8], marine sediments [9, 10], etc. are being gradually resolved using phylogenetic and gene-targeted tools.

We have also noticed that most of the world's largest oil-producing areas, encompassing the Middle East, North Africa, and Kazakhstan Block, are generally deserted or semi-deserted geographical environments. However, although a few studies have been conducted to examine the bacterial communities in deserts [11–13], our knowledge about the composition and origins of the microbial communities in these deserted or semi-deserted areas and their inter-relationships with the environments remains sparse.

In this study, the vertical distribution of the organic compounds in crude oil-contaminated Dzungharian Basin soils was resolved using a gas chromatography-mass spectrometer (GC-MS) to gain insight into natural degradation process of the crude oil in the semi-deserted soils. Subsequently, an Illumina high-throughput sequencing technique and 16S rRNA gene clone library sequencing were used to elucidate the bacterial community compositions and their vertical stratification from the surface to the bottom soil layer to answers the following questions. (i) What is the composition and structure of microbial communities in different soil layers at this site? (ii) What is the origination of the resident bacteria communities? (iii) What extent can the crude oil be degraded in the deserted and semi-deserted sites? (iv) What extent can the bacteria contribute to the natural attenuation of petroleum contamination in semi-deserted environment?

## Materials and methods

### Sampling site and environment parameter determination

The sampling sites (84^o^42′E, 45^o^36′N) are located in the northwest of Dzungharian Basin, northwest of China. It is largely a semi-deserted area in which crude oil mining was started in the 1950s. The samples were collected in the middle of October 2016. Eight sampling sites located in the Karamay oilfield which belong to Liaoning Xinke Oil Company Karamay branch. They have permitted this samples collections and also supplied necessary helps during this process. The samples mainly close to the oil wells, were collected by using augers and cores and artificially divided into different layers: 5–15 cm, 25–35 cm, and 45–55 cm. The samples were placed in glass bottles with Teflon caps and then transferred to the laboratory and stored at 4 °C for the following analysis. In the whole sampling process, there are no endangered or protected species or locations were involved in this study.

Total nitrogen (TN) concentrations of the soil samples were measured using the Kjeldahl method. Total organic carbon (TOC) in the sediment was determined by potassium dichromate oxidation spectrophotometry [14]. Total phosphorus (TP) in the sediment was determined by the ammonium metamolybdate spectrophotometric method (Lachat Method). For every trial, triplicates were conducted. The data were averaged, and the standard deviation values were statistically calculated. To count the number of bacteria, approximately five grams of fresh samples was fixed with 2% formaldehyde, diluted with deionized water to a 50-mL volume, and then rotated at room temperature for 20 min to generate a homogenate suspension. Five microliters of the suspension was stained with 4',6-diamidino-2-phenylindole (DAPI), and then the bacterial cells were counted under a fluorescence microscope (Nikon ECLIPSE 80i, Japan).

### GC-MS analysis of the organic carbon of soil samples

Approximately 5-g homogenized soil samples were extracted with 25 mL of acetone/dichloromethane solvent (v/v = 1:1) three times and then combined. The solvent volume was reduced to 5 mL using a rotary evaporator, and then approximately 25 mL of n-hexane was added to the bottle to resolve the extraction and concentrated to 5 mL. The samples were stored at −20 °C in amber glass vials for the following GC-MS analysis.

The samples were analyzed using an Agilent 7890A/5975C GC–MS machine equipped with a HP-5MS (30 m×0.25 mm×0.25 μm) capillary column. Helium (99.999%) was used as a carrier gas with a flow rate of 1.0 mL/min. An aliquot of 1 μL was injected in splitless mode at an injection temperature of 250 °C. The column temperature program was set as follows: initial temperature of 50 °C held for 2 min, 10 °C/min increase to 120 °C, followed by an increase of 4 °C/min to 310 °C (held for 15 min). The mass spectrometry conditions were as follows: EI current source of 70 Ev, mass range within 45~600 amu, voltage multiplier of 1200 V, ion source temperature of 230 °C, and interface temperature of 280 °C.

A total of 24 samples were analyzed and divided into surface soil, medium layer soil and bottom layer soil groups. The data consisting of GC–MS/SIM chromatograms for each sample were exported to AIA file format using the commercial software ChemStation (Agilent Technologies). The metabolite detector scans all single ion chromatograms (SICs) for peaks and extracts the spectra of potential compounds. NetCDF was used to retrieve relevant data (e.g., signal intensities, sample names, sample descriptions) in MATLAB 7.10.0, in which the data were pre-processed and analyzed. Based on GC/MS reference compound libraries, the compounds were identified. The process for baseline removal, chromatogram and retention time alignment, normalization, and compound integration and quantification were conducted mainly as previously described by Adhikari et al. [15].

### Total DNA extraction and T-RFLP analysis of the petroleum contaminated samples

Total DNA extraction from the petroleum-contaminated soil samples was conducted according to previous descriptions (Knaebel and Crawford, 1995; Juck et al. 2000), followed by purification using the Power Soil DNA Isolation Kit (MO BIO Laboratories). 16S rRNA genes in the total bacteria metagenomic DNA of soils samples were amplified by PCR by using the hexa-chloro derivative-labeled universal primers 26F (5’-HEX-AGA GTT TGA TCC TGG CTCAG-3’) and 1055R (5’-CAC GAG CTG ACG ACA GCC AT-3’), corresponding to *Escherichia coli* 16S rRNA positions 26–45 and 1055–1074, respectively. PCR amplification, digestion, purification and genotyping analysis by T-RFLP were conducted mainly according to the description by Yang et al. [16]. Cluster analysis and one-way ANOSIM analysis of similarity were performed with the program PRIMER V5.2 (Plymouth Marine Laboratory, Plymouth, UK). The cluster analysis was based on the similarity matrices created by comparing the relative abundance. The ANOSIM R statistic was calculated based on difference in mean ranks between and within groups.

### 16S rRNA gene clone library construction, RFLP analysis and sequencing

The process for Dzungharian basin oilfield soil layer 16S rRNA gene clone library construction, RFLP analysis, and sequencing of the representative clones were conducted mainly according to the description provided by Liang et al. [17]. Briefly, the 16S rRNA genes of the soil bacteria were amplified using 27F/1492R as the primer and soil DNA as the template. PCR products were then cloned into pMD18-T simple vector (Takara, Dalian, China) to construct the clone libraries. For each soil layer, two clone libraries were constructed. The RFLP analysis was conducted using *Hha* I and *Msp* I to digest the plasmid carrying the 16S rRNA gene, and the fingerprint of every clone was recorded. The fingerprints were then converted into a two-dimensional binary matrix through a binary scoring system (1 for the presence of a band and 0 for the absence). Based on this binary matrix, the similarity between the clone and the OTU was determined (Applied Biostatistic, Setauket, NY). A representative clone of every OTU was selected for 16S rRNA gene sequencing, and the sequences obtained from this study were deposited in GenBank under accession numbers KT353550-KT353563 and KT893461-KT893475. The 16S rRNA gene sequences of clones were aligned, and the phylogenetic analysis were carried out using the PHYLIP package (http://www.phylip.com/).

### Amplicon generation and high-throughput sequencing

The 16S rRNA gene V4 region was amplified using the barcoded specific primer set 515F (5’-GTGCCAGCMGCCGCGGTAA-3’) and 806R (5’-GGACTACHVGGGTWTCTAAT-3’) with total DNA from the petroleum contaminated soil layers as the template. PCR products from different soil layers were then employed for library construction and sequencing. Sequencing libraries were generated using the TruSeq DNA PCR-Free Sample Preparation Kit (Illumina, USA) following the manufacturer’s recommendations, and then the libraries were sequenced on an IlluminaHiSeq 2500 platform.

Paired-end reads were sorted and assigned to samples based on their unique barcodes. Paired-end reads were merged using FLASH (V.1.2.3, http://ccb.jhu.edu/software/FLASH/). Quality filtering of the raw reads was performed under specific filtering conditions to obtain high-quality clean reads according to the QIIME (v1.7.0, http://qiime.org/index.html) quality-controlled process. The reads were compared with the reference database (Gold database, http://drive5.com/uchime/uchime_download.html) using the UCHIME algorithm (UCHIME algorithm, http://www.drive5.com/usearch/manual/uchime_algo.html) to remove chimera sequences. The clean sequences were deposited in the NCBI Sequence Read Archive (SRA) database under BioProject No. PRJNA386950 (SRP107616).

### OTU determination, taxonomic annotation and diversity analysis

The operational taxonomic unit (OTUs) determination was performed using Uparse software (Uparse v7.0.1001, http://drive5.com/uparse/) on effective reads of every sample. Sequences with a similarity higher than 97% were assigned to the same OTUs. A representative sequence for each OTU was screened for further annotation. For each representative sequence, the GreenGene Database (http://greengenes.lbl.gov/cgi-bin/nph-index.cgi) was used based on the RDP classifier (v2.2, http://sourceforge.net/projects/rdp-classifier/) to annotate the taxonomic information. To examine the phylogenetic relationship of different OTUs, multiple sequence alignment was conducted using MUSCLE software (v3.8.31, http://www.drive5.com/muscle/).

The indices, Chao1 estimator (http://www.mothur.org/wiki/Chao), ACE estimator (http://www.mothur.org/wiki/Ace), Shannon index (http://www.mothur.org/wiki/Shannon) and Simpson index were selected to identify community diversity, richness and evenness, and Good’s coverage index was selected to characterize the sequencing depth. All these indices in our samples were calculated with QIIME (v1.7.0) and displayed with *R* software (v2.15.3).

### Clustering and PCoA analyses

The bacterial community structures in different layers were compared using weighted UniFrac based on the phylogenetic relationship of representative reads from different layers, and the weighted UniFrac was calculated by QIIME software (v1.7.0). The sequences of representative reads from all the sampling were used to reconstruct multidimensional data. The principal coordinate analysis (PCoA) was performed to determine the principal coordinates and visualize complex, multidimensional data. PCoA analysis was displayed using the WGCNA package in R software (v 2.15.3). A weighted pair-group method with arithmetic mean (UPGMA) clustering was performed as a type of hierarchical clustering method to interpret the distance matrix using average linkage and was conducted with QIIME software (v 1.7.0). The correlation between the bacterial communities and environmental parameters was evaluated by canonical correspondence analysis (CCA) using the software Canoco5 (http://www.canoco5.com/). The reference sequences were extracted from GenBank under accession numbers for water column of the Clarion-Clipperton fracture zone in the Pacific Ocean (SRR1980879, SRR1980893 and SRR1980904), estuary water (ERX1529128, ERX1529080), environmental soil samples (SRR1554795, SRR1554978), and crude oil (ERR958514, ERR1039276).

## Results

### Environmental parameters of the sampling sites

**Fig 1:**
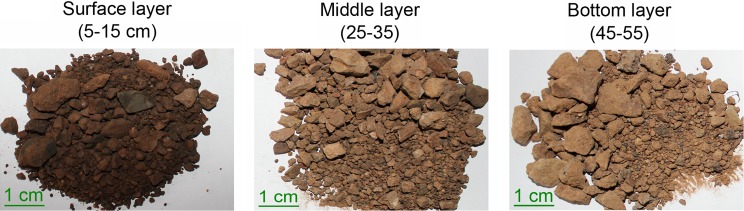
Phenotypes of crude oil-contaminated soil samples. Soil columns from the surface down to a depth of approximately 55 cm were divided into three layers. The green dash indicates one centimeter.

In our previous field investigation, the crude oil could penetrate from the surface soil down to a soil depth of 55 cm (Fig 1). Thus, from the surface to a depth of 55 cm, we artificially divided the soil cores into three layers: surface layer (5–15 cm), middle layer (25–35 layer), and bottom layer (45–55 cm). As shown in Table 1, the content of TOC, TN and TP in the uncontaminated soils gradually increased from the oligotrophic surface layer to the bottom layer. In the petroleum-contaminated soils, the TOC in the surface layer samples reached 48.39±0.12 mg/g soils, which was significantly higher than in the uncontaminated counterpart (0.58±0.15 mg/g), indicating serious petroleum pollution. In the middle layer, the TOC value decreased to 20.28±0.92 mg/g, and in the bottom layer, to 2.31±0.65 mg/g, which was slightly higher than the uncontaminated counterpart (1.86±0.75 mg/g). Because the contaminated crude oil might contain certain amount of nitrogen and phosphates, the TN and TP of the petroleum-contaminated soil layers were higher than the uncontaminated soils. TN and TP in the petroleum-contaminated surface layer were 0.65±0.02 mg/g and 0.77±0.006 mg/g, respectively. In the middle layer, the value decreased to 0.43±0.15 mg/g and 0.65±0.008 mg/g. As determined by the DAPI staining method, the oligotrophic semi-deserted soils still harbored certain amounts of bacteria. Especially in the bottom layers of both contaminated and uncontaminated soils, the numbers of bacteria reached 2.5×10^7^ and 2.42×10^7^ per gram soil, respectively, which were significantly higher than in the surface layer and middle layer.

**Table 1:** Environmental parameters of soil layers in the sampling sites.

Soil layer		Petroleum-contaminated soils					Uncontaminated soils			
	TOC (mg/g)	TN (mg/g)	TP (mg/g)	TOC/TN	Number of bacteria	TOC (mg/g)	TN (mg/g)	TP (mg/g)	TOC/TN	Number of bacteria
Surface layer (5–15 cm)	48.39±0.12	0.65±0.02	0.77±0.006	74.45	2.27106	0.58±0.15	0.25±0.02	0.40±0.04	2.32	2.47106
Middle layer (25–35 layer)	20.28±0.92	0.43±0.15	0.65±0.008	47.16	4.55106	0.95±0.42	0.35±0.04	0.52±0.06	2.72	4.25106
Bottom layer (45–55 cm)	2.31±0.65	0.67±0.02	0.79±0.03	3.45	2.5107	1.86±0.75	0.56±0.02	0.65±0.05	3.32	2.4207

### GC-MS analysis of organic compounds in petroleum contaminated samples

**Fig 2:**
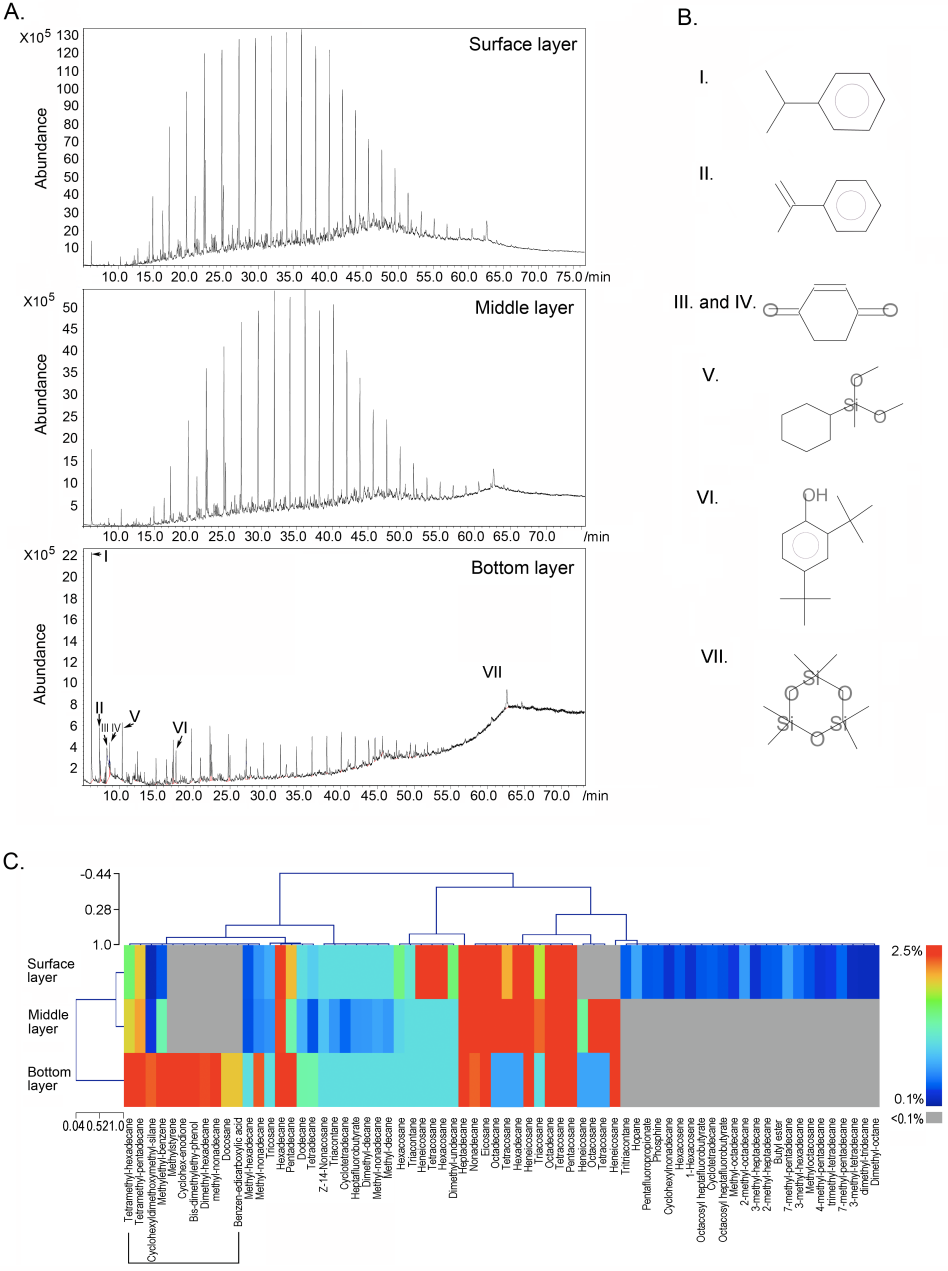
GC-MS analysis of the organic compounds in the three layers of petroleum-contaminated soil samples. A. GC chromatograms of the compounds. B. Molecular structures of the aromatic compounds and heterocyclic compounds specifically denoted by arrows in the GC chromatograms. C. The heatmap indicates the profile of organic compounds with a normalized content >0.1%.

The GC-MS method was used to analyze the variation in the petroleum-derived components in different soils layers. From the surface layer down, to the middle layer, and to the bottom layer samples, approximately 370, 300 and 230 types of components were identified and structurally resolved, respectively. The components with a normalized peak area (area%) greater than 0.1 were selected for cluster analysis among different layer samples (Fig 2). As shown in Fig 2A, alkanes were the main components of petroleum contaminants in the surface and middle layers, with carbon chain lengths ranging from octane to tritriacontane. Due to the efficient penetration of crude oil in the loose texture of soil layers, the spectra of components of surface and middle layers were very similar. Degradation of petroleum contaminants occurred in the bottom layer. In this layer, the crude oil was almost totally degraded into short-chain compounds, with the spectrum of organic components in this layer distinguished from the upper layer (Fig 2A).

A more detailed view of the components of every soil layer revealed that during the process of degradation of crude oil, in which the long-chain alkanes were degraded into short-chain alkanes and other types of small molecule compounds, two types of components, aromatic compounds and heterocyclic compounds, were efficiently enriched in the soils. As represented by 1-methylethyl-benzene, methylstyrene, 1,4-cyclohex-2-enedione, 2,5-bis(1,1-dimethylethyl)-phenol, and especially the 1-methylethyl-benzene increased from 0.1% in the surface layer to a significantly greater level of 11.4% in the bottom layer (Fig 2B and 2C). We further examined 79 types of aromatic and heterocyclic compounds, which accounted 34.4% of all identified compounds in the bottom layer (Fig 2C).

### T-RFLP clustering of bacterial communities from different sampling sites

**Fig 3:**
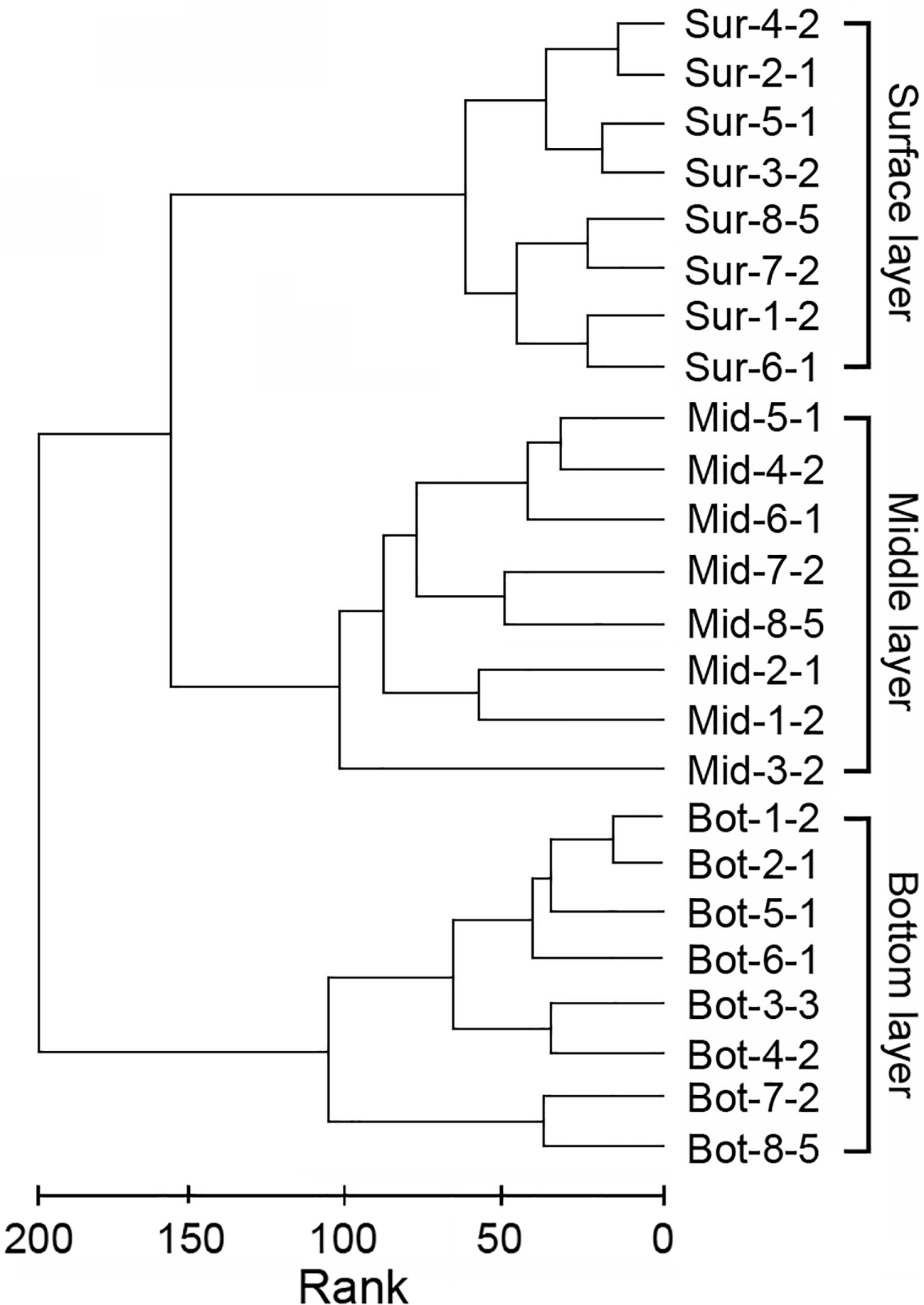
Dendrogram showing the similarity of bacterial communities revealed by T-RFLP analysis of the 16S rDNA from different layers of the petroleum-contaminated soils. Samples were collected from eight sites, and three layers per sites were analyzed.

In total, 24 soil samples collected from eight sites (three layers per site) were subjected to T-RFLP analysis to determine the bacterial community profiles. A total of 225 T-RFs of different sizes were obtained from these petroleum-contaminated soil samples. Cluster analysis based on the fingerprints of these samples indicated that the bacterial communities in petroleum-contaminated samples could be divided into three clusters according to their vertical stratification with generally high values of *R*>0.85 (Fig 3). Samples from the same soil layer, regardless of the sampling site, shared high similarity in their bacterial communities, generally with *R*<0.25 in the ANOSIM comparison. Thus, the T-RFLP profiles of the surface layer samples from different sites were grouped into one cluster and diverged from the middle and bottom layer samples. The latter two layers were also divergent from each other (*R* = 0.95, *p*<0.05) according to their vertical stratification.

### Diversity and species richness of bacterial communities

**Fig 4:**
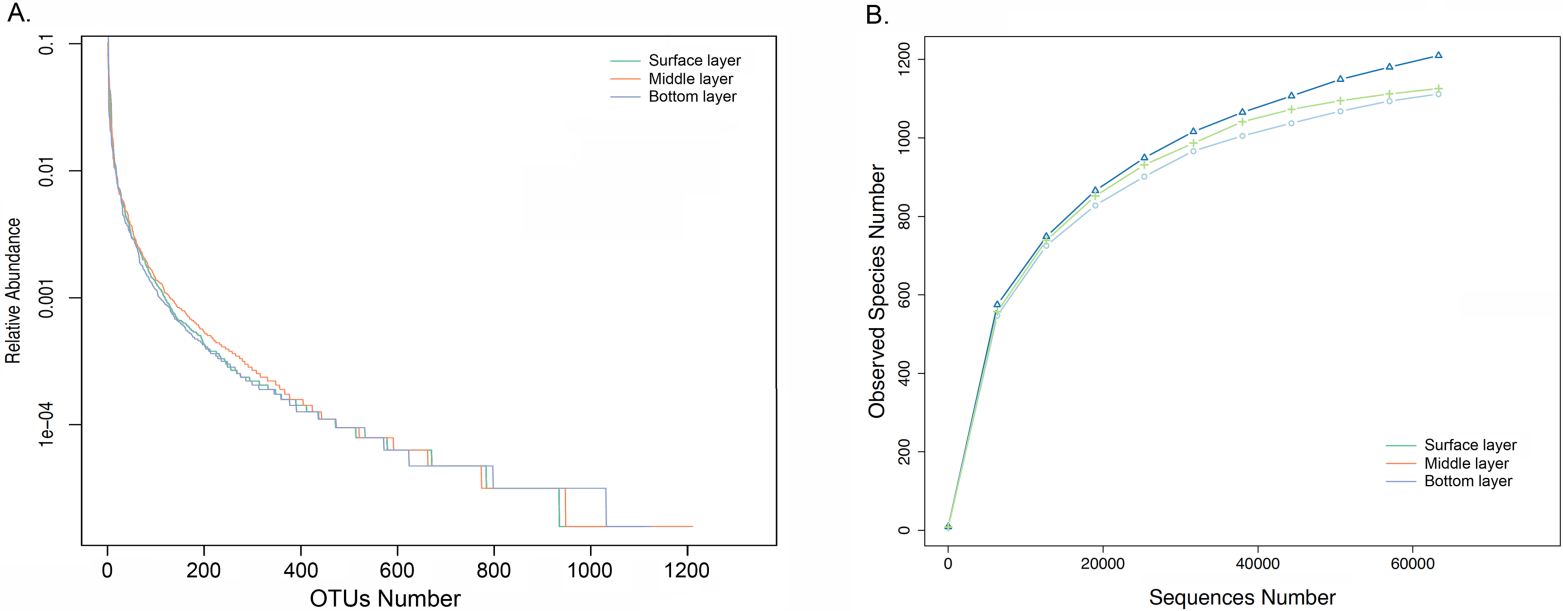
**The rank-abundance curves (A) and rarefaction curves (B) based on the bacterial OTUs determined at 97% similarity of the sequences indicating the diversity, richness and coverage of the bacterial communities in the three layers of petroleum-contaminated soils**.

Approximately 300 000 raw sequences of the 16S rRNA gene spanning the hypervariable V4 region were obtained from the soil samples. After screening out noise, poor-quality reads and chimera sequences, approximately 69000 sequences in the surface layer, 112000 sequences in the middle layer, and 63000 sequences in the bottom layer, with an average length of 252 bp, were selected for subsequent analyses (S1 Fig). As indicated by the Good_coverage index (>0.996), these sequences provided good coverage of the bacterial communities in the samples. Compared with the surface layer (1112) and bottom layer (1126), the middle layer harbored the greatest number of OTUs (1210). The middle layer also contained the most diverse bacteria compared the crude oil heavily polluted surface layer and bottom layer, with Shannon, Chao1 and ACE indices that were significantly higher than for the other two layer samples. In contrast, the bottom layer harbored the greatest amount of bacteria (Table 2). Coincident with the Good_coverage index, the rarefaction curves generated from the OTUs defined at 97% similarity further indicated that the bacterial sequences well represented the bacteria communities as the curves were approaching plateaus when the sequence number exceeded 60,000. Rank-abundance curves indicated that all samples contained relatively low proportions of highly abundant bacteria (less than 120 species with a relative abundance >0.001), and a majority of the sequences belonged to rare species of microorganisms represented by only a small proportion of the sequences (relative abundance <0.001). As indicated by the span of the curve on the horizontal axis, the middle-layer samples contained a much greater abundance of rare sequences than the other two layers (Fig 4).

**Table 2:** Diversity indices of the bacterial communities in the three soil layers.

Sample name	Taxon sequences	Good_coverage	Number of OTUs (97% similarity)	Shannon	Simpson	Chao1	ACE
Surface layer	69273	0.997	1112	6.694	0.972	1215.638	1221.806
Middle layer	112723	0.996	1210	6.884	0.973	1406.874	1418.509
Bottom layer	63328	0.998	1126	6.276	0.941	1145	1171.075

### Taxonomic assignment of bacterial reads from different soil layers

At a confidence threshold of 50%, approximately 95% of the taxon sequences, or 245,325 of 257,685 qualified reads, were assigned to known phyla. Altogether, 23 bacterial phyla with a ratio higher than 0.01% in the total reads were recovered from our samples (S2 Fig). More than 95% of the bacterial reads from the samples were affiliated with seven dominant phyla (sub-phyla): *Gammaproteobacteria* (50.0%), *Alphaproteobacteria* (19.4%), *Betaproteobacteria* (4.2), *Actinobacteria* (17.0%), *Firmicutes* (3.3%), *Plantomycetes* (1.6%), and *Bacteroidetes* (1.0%). The phyla *Chloroflexi* (0.7%), *Thermi* (0.6%), *Gemmatimonadetes* (0.5%), *Acidobacteria* (0.15), TM7 (0.1%) and WPS-2 were minor groups, with ratios in the whole bacterial community less than 1%.

The divergence among communities from the surface layer down to the bottom layer was observed at the phylum level. As shown by S2 Fig, the surface layer contained greater amounts of *Alphaproteobacteria* (23.7%), but the middle layer harbored more *Actinobacteria*, *Planctomycetes*, and *Thermi*. In the bottom layer, *Gammaproteobacteria* (56.90%) and *Firmicutes* (4.1%) displayed the highest ratio compared with the other layers.

**Fig 5:**
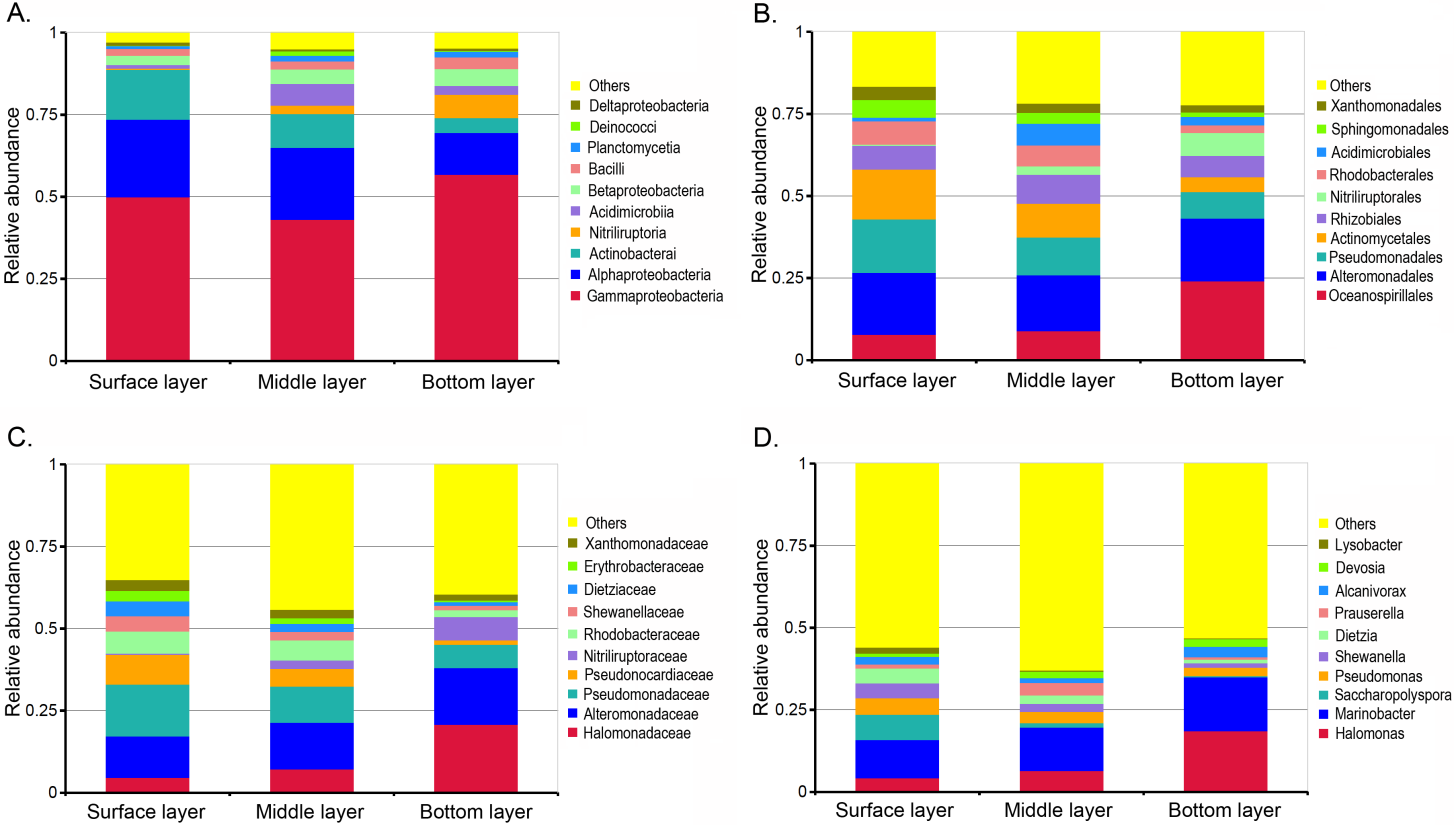
**Relative abundance of the top 10 groups classified at the level of sub-phylum and class (A), order (B), family (C) and genus (D) in communities from different soil layers**.

To conduct a more detailed analysis of the composition of the communities in the soil column, we classified all the reads that had been assigned into a phylum down to the class, order, family and genus levels. The vertical stratification of bacterial communities was further evidenced in the major cluster identified for all layers. To simplify the comparison among different layer and provide more concise descriptions, we mainly compared those groups classified in the top 10 of the ratio list among whole bacterial communities at different levels of classification (Fig 5).

As shown in Fig 5A and 5B, the surface-layer samples harbored a higher ratio of *Actinomycetales* (15.2%), *Pseudomonadales* (16.4%), *Rhodobacterales* (7.0%), *Sphingomonadales* (5.4%), and *Xanthomonadales* (4.0%), but the bottom-layer samples were characterized by a large proportion of *Oceanospirillales* (24.2%), *Alteromonadales* (19.1%), and *Nitriliruptorales* (7.0%). The middle layer harbored a relatively higher amount of *Rhizobiales* (8,8%) and *Acidimicrobiales* (6.5%). At the family level, we found that *Halomonadaceae* (20.9%), *Alteromonadaceae* (17.2%), and *Nitriliruptoraceae* (7.0%) were especially enriched in the bottom layer, and *Pseudomonadaceae* (15.8%), *Pesudonocardiaceae* (9.0%), *Shewanellaceae* (4.6%), and *Dietziaceae* (4.5%) had a higher ratio in the surface layer community (Fig 5C). The classified top 10 genera found in petroleum-contaminated soils were *Halomonas*, *Marinobacter*, *Saccharopolyspora*, *Pseudomonas*, *Shewanella*, *Dietzia*, *Prauserella*, *Alcanivorax*, *Devosia*, and *Lysobacter*. Coincident with observed order level, two major genera, *Halomonas* and *Marinobacter*, were enriched in the bottom layer with contents of 18.7% and 16.5%, which were significantly higher than in the surface layer and middle layers. In contrast, the genera *Saccharopolyspora*, *Pseudomonas*, *Shewanella*, *Dietzia* and *Lysobacter* were more abundant in the upper layers (Fig 5D).

### Bacterial community divergence and their relationship with environmental factors

We compared the similarity and divergence of the bacterial communities in three petroleum-contaminated soil layers. As shown by Venn maps, the surface layer, middle layer and bottom layer had approximately 835, 786 and 947 common OTUs between two of them, respectively (S3 Fig). Among all the communities, 714 OTUs were commonly present in the three communities. In addition, the communities from each layer had distinct OTUs: 205, 142, and 107 OTUs were specific to the surface, middle and bottom layer communities, respectively (S3 Fig).

**Fig 6:**
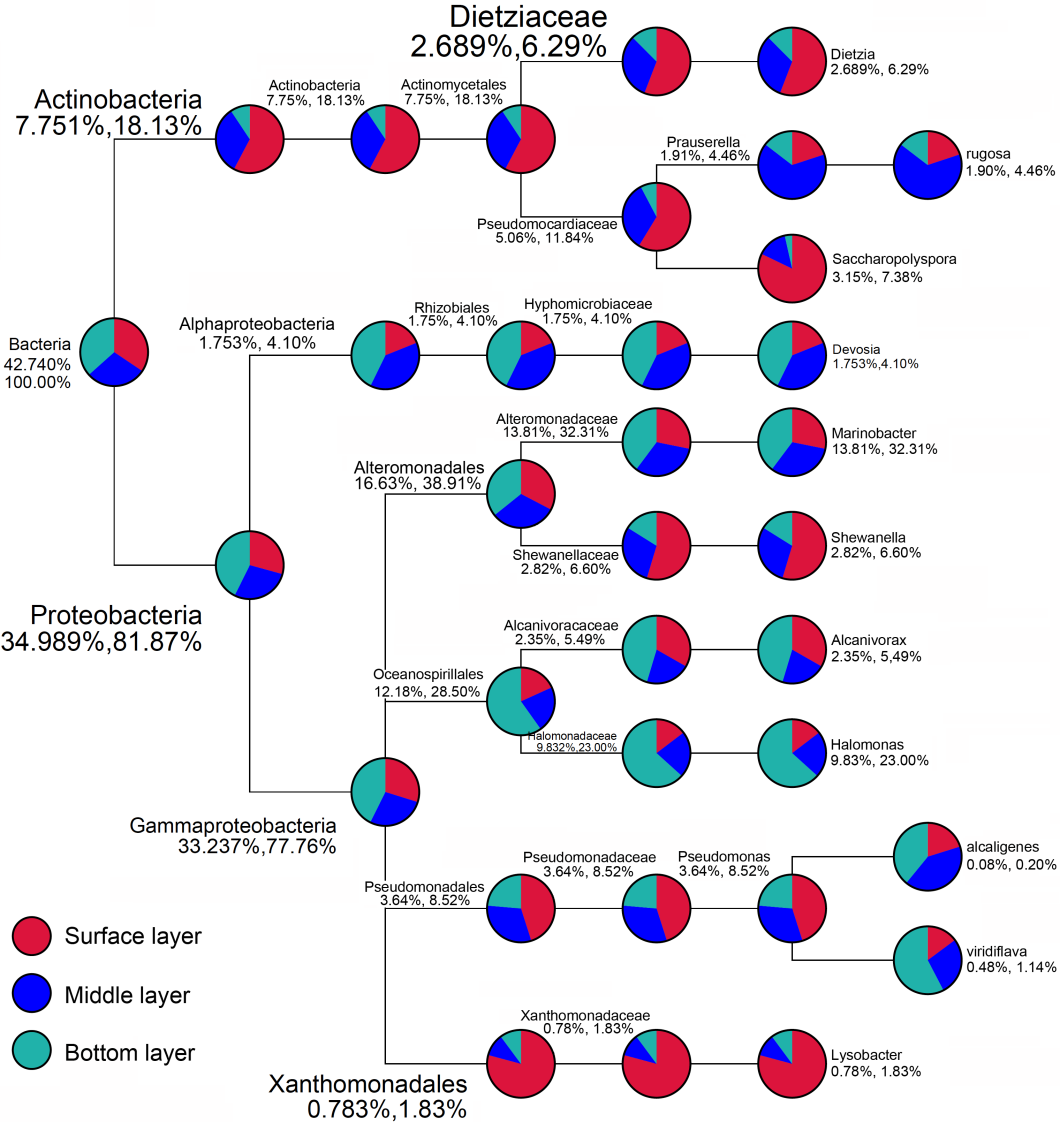
Taxonomic tree of the top 10 genera among all the bacteria communities. The pie chart indicates the ratio of communities from different soil layers. The ratio of selected taxonomic units among all bacteria communities and the ratio in the upper level taxonomic unit are listed under the taxonomic name.

**Fig 7:**
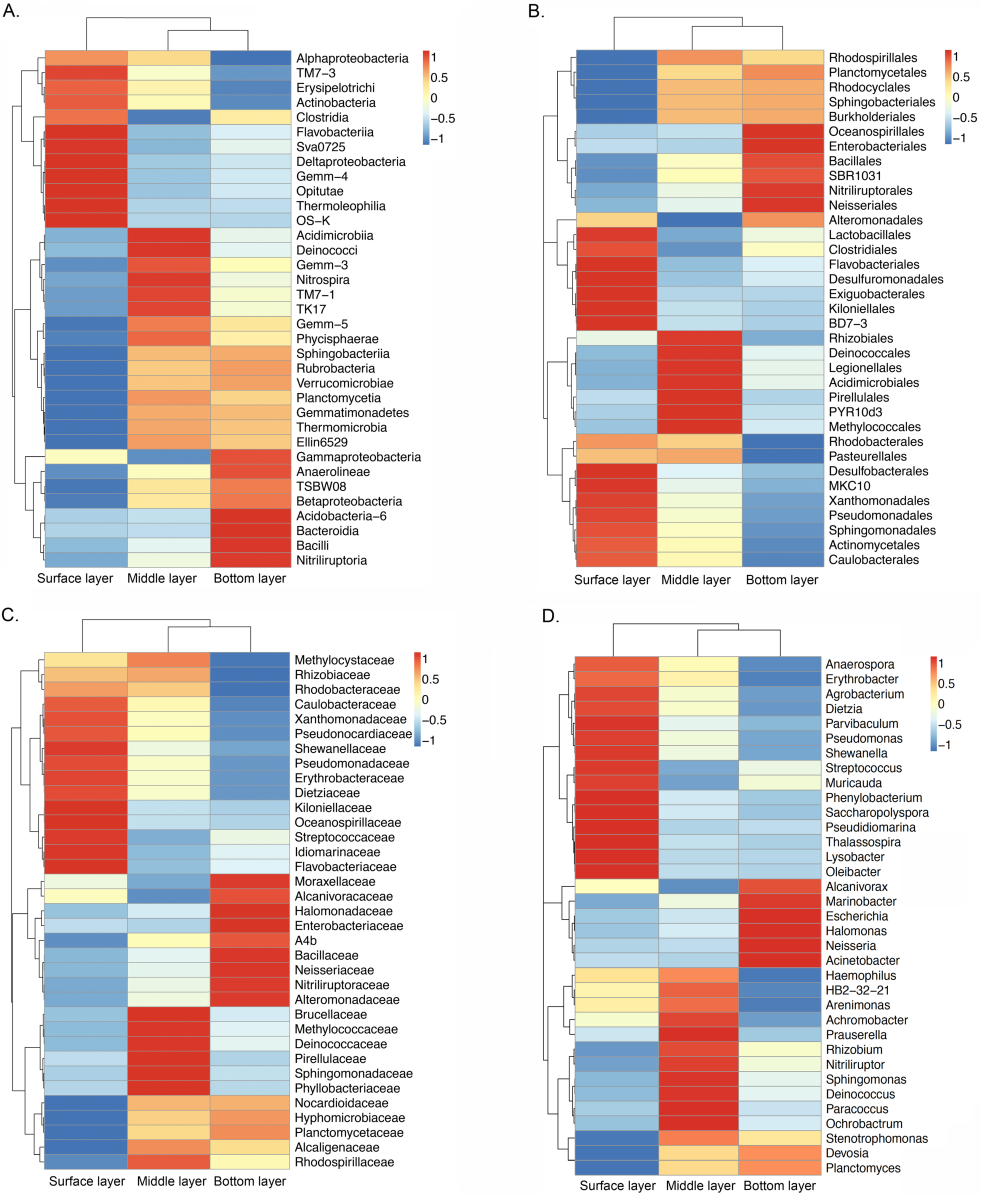
**Heatmap showing the composition of the communities from the soil layers classified at the level of class (A), order (B), family (C) and genus (D)**. Those units with abundant lists in the top 35 of the whole community were selected.

Clustering analysis among bacterial community was conducted at the phylum down to genus level (Fig 6). As shown by the heatmaps, the community compositions further exhibited vertical stratification among bacterial communities from the surface layer down to the bottom layer. The surface layer was characterized by *Lysobacter* (*Xanthomonadales*), *Thalassospira* (*Coscinodiscophyceae*), *Pseudomonas* (*Pseudomonadales*), *Shewanella* (*Alteromonadales*), *Streptococcus* (*Bacilli*), *Muricauda* (*Flavobacteria*), among others, while the bottom layer was characterized by *Halomonas* (*Oceanospirillales*), *Marinobacter* (*Alteromonadales*), *Acinetobacter* (*Pseudomonadales*), and *Alcanivorax* (*Oceanospirillales*) from the *Gammaproteobacteria*. The middle layer, however, was enriched for *Paracoccus* (Rhodobacterales), *Sphingomonas* (Sphingomonadales), *Nitriliruptor* (*Nitriliruptorales*), *Prauserella* (*Pseudonocardiales*), etc. (Fig 7A, 7B, 7C and 7D). As shown by the hierarchical clustering analysis and weighted UniFrac analysis, the community similarity of every soil layer was not stable (S4 Fig). PCoA analysis showed that communities from three layers were separately distributed into different dimensions, revealing the distinct vertical stratification of the communities from different soil layers in the contaminated soils (S4 Fig).

CCA analysis was conducted to examine the bacterial communities, especially the top 10 identified genera, in response to petroleum-contaminated environmental variables (S5 Fig). As shown in Table 1, the heavy crude oil polluted surface layer characterized as having the highest TOC and TOC/TN values was distributed in the positive region by CCA2, while the bottom layer with the lightest contamination characterized with the lowest TOC value and higher TN and TP values was distributed in the positive region by CCA1. The spatial distribution of *Halomonas* and *Marinobacter*, the major identified genera in the communities, was close to the bottom sites and showed a positive correlation (*r*-value<0.25) with TN and TP. The genera *Lysobacter*, *Pseudomonas*, *Thalassospira*, *Shewanella*, *Streptococcus*, among others, which were close to surface sites, had positive correlations with TOC and TOC/TN (*r*-value = ~0.5), reflecting their adaptability in organic nutrient-rich environments. In contrast, *Prauserella* and *Lysobacter* remained almost neutral with respect to nutrients close to the middle layer samples.

### Community analysis of bacteria in Dzungharia Basin with reference sites

**Fig 8:**
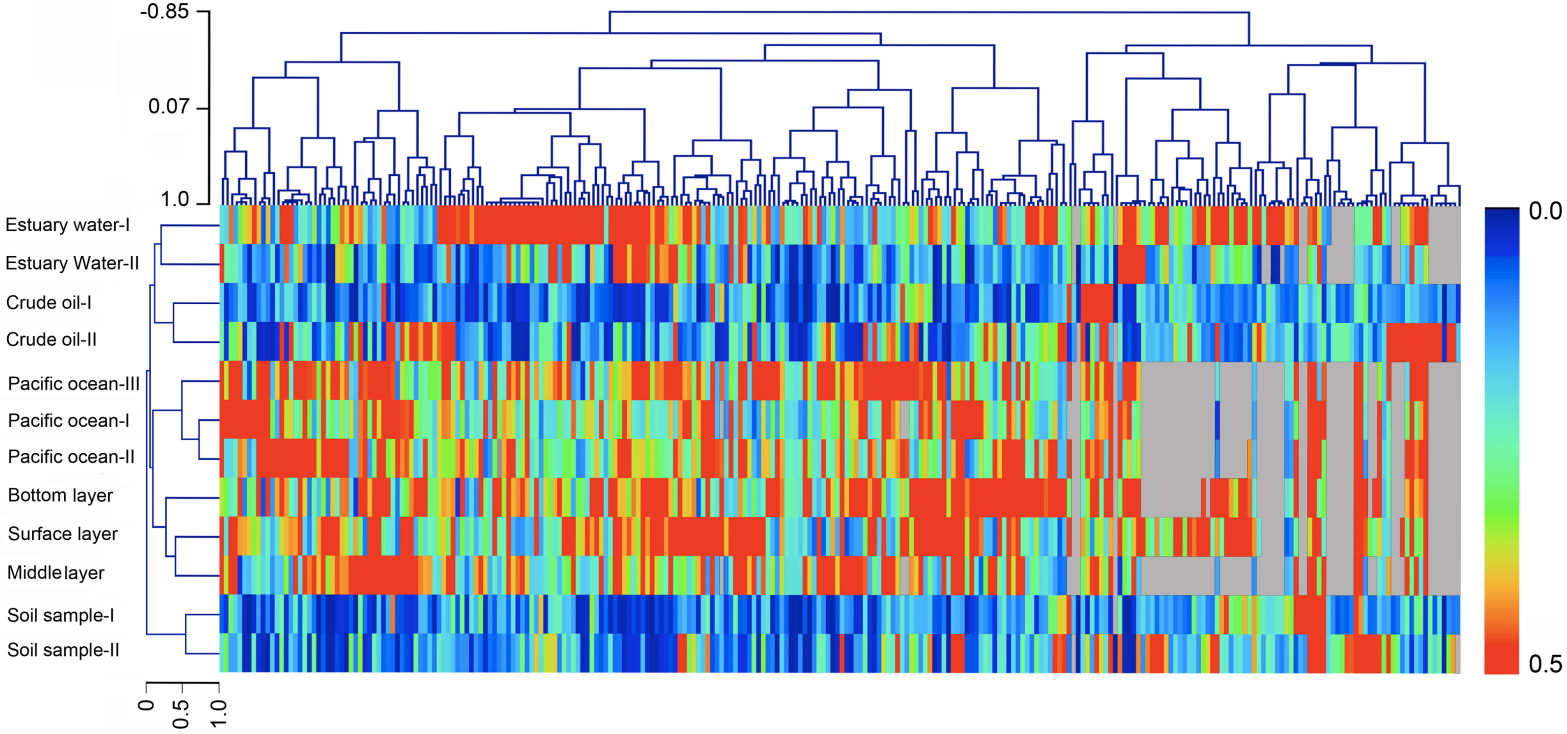
Heatmap showing the hierarchical clustering analysis of the communities from different ecological sites. Pearson correlation distance metric and average linkage clustering methods were used to construct HCL trees of the samples and OTUs determined at 95% similarity. The reference sequences were extracted from the water column of the Clarion-Clipperton fracture zone in the Pacific Ocean (SRR1980879, SRR1980893 and SRR1980904), estuary water (ERX1529128, ERX1529080), environmental soil samples (SRR1554795, SRR1554978), and crude oil (ERR958514, ERR1039276).

As revealed above, bacterial communities, especially the bottom soil layer harbored a large numbers of *Halomonas* and *Marinobacter* strains. This finding suggested that the bacterial communities in this region might have a marine origin. Considering that human activity and petroleum contamination might also affect the community composition of the bacteria, we compared Dzungharia Basin communities with those from different ecological environments. The reference sequences were extracted from the water column of Clarion-Clipperton fracture zone in the Pacific Ocean (SRR1980879, SRR1980893 and SRR1980904), estuary water (ERX1529128, ERX1529080), typical soil samples (SRR1554795, SRR1554978), and crude oil (ERR958514, ERR1039276). A hierarchical clustering analysis was conducted among these communities (Fig 8). As shown in Fig 8, the community in Dzungharia Basin was clearly closely related to the communities with a marine origin and divergent from the crude oil, soil and estuary water communities.

**Fig 9:**
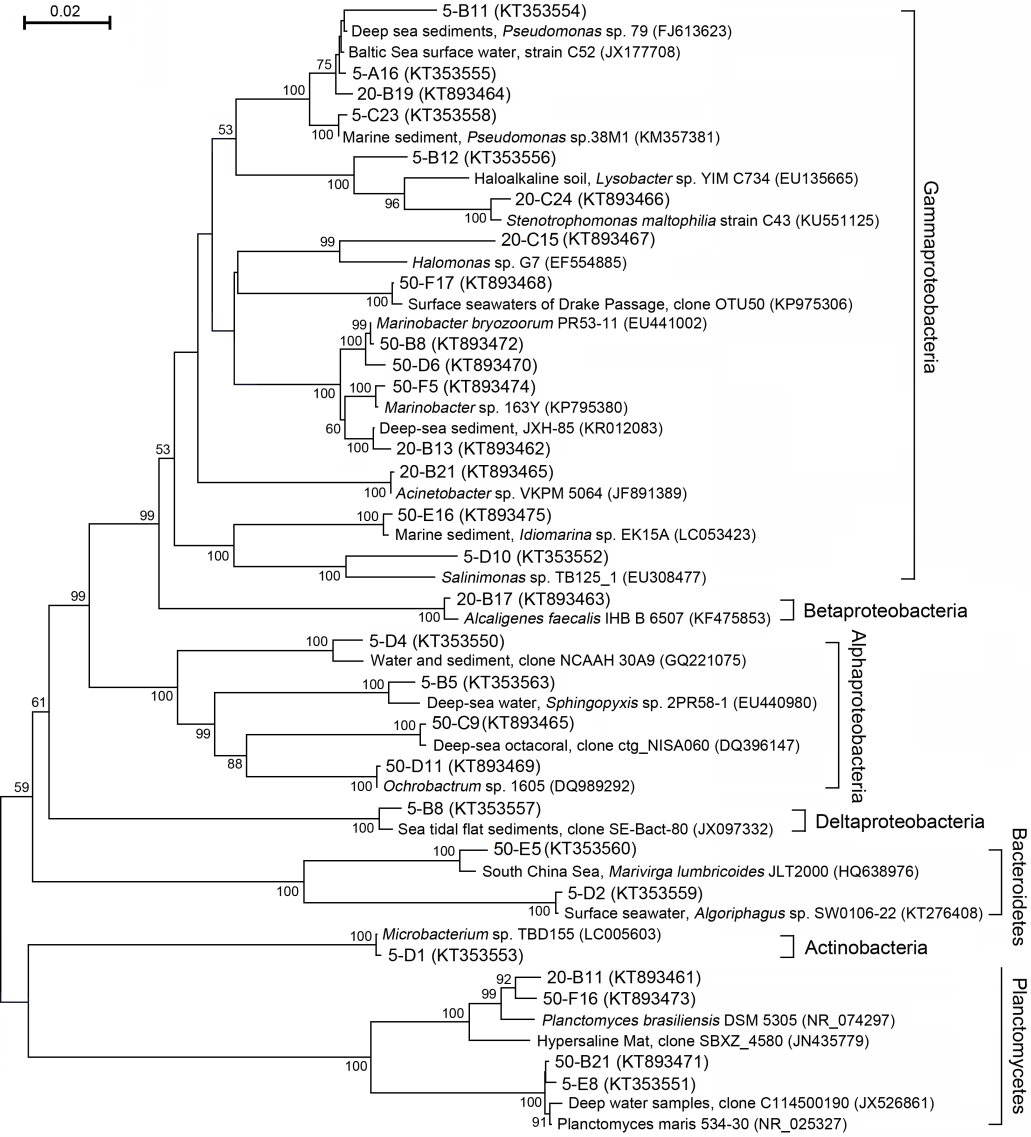
Neighbor-joining phylogeny tree of 16S rRNA gene sequences of the representative clones of the libraries for three soil layers. Clone from the environmental communities were selected as the references. Bootstrap values (n = 1,000) greater than 50% are indicated in the subclusters that were specifically present in Karamay ecological environments.

Bacterial 16S rRNA gene (approximately 1465 bp) clone libraries of these soil layers were constructed. After RFLP clustering, the representative clones of every OTU were sequenced [17] (Liang et al. 2016). A phylogenic tree was constructed based on the 16S rRNA gene sequences of the representative clones and the reference sequences from different geographical sites (Fig 9). Coincident with the classification results based on the Illumina high-throughput sequences, *Gammaproteobacteria* still represented the majority of the communities. Among this cluster, clones from *Marinobacter*, *Halomonas*, and *Pseudomonas* were frequently present in these communities. Clones in this cluster were generally grouped with clones from deep-sea sediment, surface seawater, marine coastal sediment, etc. Even the clones in *Alphaproteobacteria*, *Bacteroidetes*, and *Planctomycetes* were also closely grouped with reference clones and pure cultures from marine sediment, surface seawater, etc.

## Discussion

### Petroleum component resolution

GC-MS was an efficient and powerful measure for analyzing and resolving environmental pollution. This method has been gradually used to investigate the biodegradation of petroleum in a sub-arctic site to identify the source of pollution [18], tracing the temporal and spatial variability of aliphatic hydrocarbons and linear alkylbenzenes in a subtropical estuary [19], addressing petroleum degradation in deep water [15], etc. However, to date, there has been no GC-MS-coupled ecological study on petroleum-contaminated deserted and semi-deserted environments. In this study, we successfully used the GC-MS method to resolve and determine the detailed structure of the organic compounds in the different layers of petroleum-contaminated semi-deserted soils. Due to the high resolution of GC-MS, both major components such as alkanes and trace components such as aromatic compounds and heterocyclic compounds were all assessed in the contaminated soil. In total, from the surface layer, to the middle layer, and down to the bottom layer, approximately 370, 300, and 230 types of components were identified and structurally resolved, respectively. According to the quantitative comparison between every layer, we also observed that aromatic compounds and heterocyclic compounds were enriched in the bottom layer during the process of degradation (Fig 2). With advantages such as a simplified procedure, easily accessible database for structural determination, and economic benefits, this method could be recommended as a routine method for future assessments of the environmental parameters in organic compound-contaminated environments.

### Natural attenuation of crude oil contaminants in soils

The climate of this semi-deserted and deserted Dzungharia Basin changes dramatically in this area. In winter, due to invasion of the Siberia cold current, the lowest temperature can reach −35 °C. In summer, due to the long days of sunlight and arid climate, the temperature can reach 45 °C and the land surface temperature can reach approximately 80 °C (http://tianqi.2345.com/xinjianglvyou/). As shown in Table 1, the surface layer was an oligotrophic environment, while the 50-cm-deep layer showed a significantly increased nutrient content, which supplied a suitable niche for habitation by microorganisms.

Although a variety of bioengineering measures have been adapted to remediate petroleum-contaminated environments [5–7], most of the world’s largest oilfields are located in deserted or semi-deserted geographical environments, such as the Middle East and North Africa. In these areas, remediation of the contaminated soil will depend more on the natural attenuation of the petroleum compounds [11, 20, 21]. The natural degradation of crude oil generally encompasses three aspects: physical, chemical and biological degradation. Compared with the physical and chemical measure for remediation of petroleum-contaminated ecological sites, the ubiquity of hydrocarbon-utilizing microorganisms may play a role in this process, but to what extent this *in situ* biodegradation contributes to the natural attenuation of petroleum contamination remains to be discussed.

In this study, the GC-MS results clearly showed that the organic compound spectrum in the surface and middle layer were very similar to crude oil. A harsh environment, such as the arid climate, long days of sunlight, extreme temperature, etc., had a weaker effect on the degradation of crude oil (Fig 2A). In the bottom layer, however, most of the crude oil components had been degraded. Compared with the surface and middle layers, the bottom layer had a milder environment and was exposed to fewer climactic disturbances. In these sites, there was a significant enrichment for many bacteria that might participate in the degradation of petroleum (Fig 2): *Alcanivorax*, a very important oil-degrading organisms [22]; *Dietzia*, a member of which is generally adapted for oil degradation [7]; *Acinetobacter*, an important soil organism, some members of which contribute to the mineralization of aromatic compounds [23]; and *Marinobacter*, a genus of Proteobacteria found in seawater, many species and strains of which can degrade hydrocarbons [24]. Considering that the greatest number of bacteria inhabited the bottom soil layer, we suggest that the natural attenuation of crude oil contaminants in the soils is a bacterial-mediated process that occurs in deeper soil layers.

A detailed view on the components of the petroleum contaminants, especially in the bottom layer, showed that crude oil was degraded into short chain compounds; two types of compounds, aromatic compounds and heterocyclic compounds, were significantly enriched. In total, we have identified 79 types of aromatic compounds and heterocyclic compounds in the bottom layer and up to 34.4% types of all identified compounds. The representative 1-methylethyl-benzene significantly increased from 0.1% in the surface layer to 11.4% of the total amount of compounds. As observed in our and previous studies [25], although biological degradation of the crude oil is the most promising process in the natural environment, our study further indicates that ecological remediation of crude oil-contaminated semi-deserted and deserted soil remains a chronic process.

### Origination, vertical stratification of bacterial communities and their relationship with the environment

The association between microorganisms and ecological niches is often considered interactive. Ecological sites serve as a shelter and offer a consistent nutrient supply for bacteria; in turn, bacteria alter the environment at microcosmic or macrocosmic scales. Generally, the geographical origin and parallel transmission (or invasion) have been suggested to explain the establishment of the microorganism community [26, 27]. The geographical origin of specific microbes is a fundamental factor that affects the structure of communities. In this scenario, an ecological niche might be colonized by an ancestral strain, undergo vertical transmission, and then evolve to become specific to that ecological niche. Phylogeny analysis revealed that soils in Dzungharia Basin were enriched for marine-originated bacteria. *Halomonas* (*Oceanospirillales*), *Marinobacter* (*Alteromonadales*) and *Alcanivorax* (*Oceanospirillales*) made up high proportions of the whole communities, typically in the bottom layer. Community comparisons of bacteria from different ecological environments showed that for this inner land, deserted and semi-deserted region closely clustered with those from marine environments (Fig 6). Clustering analysis based on the clone libraries further revealed that these representative clones from Dzungharia Basin were generally clustered with the reference clones with a marine environment source (Fig 7).

Environmental introduction and transmission could also affect and shape the structure of microbial communities. Microorganisms from outer environments can be transmitted into ecological niches and then be selectively retained in local niches. When the microbes encounter favorable conditions and outcompete other potential colonizers, they multiply and gradually establish themselves within local niches. Thus, the establishment of this relationship relies on the interaction between the microbe and its environmental partners [28, 29, 30]. Generally, the bacterial communities in these petroleum-contaminated soils had three potential sources: 1) bacteria indigenous to the soil itself, which might be traced to ancient environments; 2) invasion by bacterial communities from adjacent areas by precipitation of sand transfer by wind, water and rain, or human and animal activity; and 3) indigenous microorganisms of crude oil [28]. For every soil layer, disturbances caused by the outer environments were different. Thus, vertical stratification of the bacterial communities among petroleum-contaminated soil layers was clearly observed in this study. As described in the “Results” section, the surface layer samples harbored a higher ratio of *Actinomycetales*, *Pseudomonadales*, *Rhodobacterales*, *Sphingomonadales*, and *Xanthomonadales*, but the bottom layer samples, which had the least disturbance, were characterized by a large proportion of *Oceanospirillales*, *Alteromonadales*, and *Nitriliruptorales*. The middle layer harbored relatively greater amounts of *Rhizobiales* and *Acidimicrobiales* (Figs 3 and 4).

Comprehensively, oligotrophic Dzungharia Basin soils harbor abundant bacteria. Among these soil layers, the bacteria in the middle layer have the highest diversity and richness, and the bottom layer has the largest number of bacteria. In evaluations of the interactions among the bacteria, soil geochemical properties, and crude oil contamination, bacterial communities in this region exhibited distinct vertical stratification from the surface layer down to the bottom soil layer. As represented by the bottom soil layer communities, bacteria in this region might have a marine origin or even the biological relics of an ancient ocean environment. The natural attenuation of petroleum contaminants in semi-deserted and deserted soils was mainly a microorganism-mediated degradation process in the 45-55-cm-deep layer of soil. During this process, aromatic and heterocyclic compounds were significantly enriched in the soil.

## Supporting information

S1 Fig Statistics of the number of the reads of every soil layers after sorting and classification. The number of OTUs defined at the level of 97% similarity.
(TIF)

S2 Fig Composition of bacteria in the communities from crude oil contaminated soil layers at the phylum level. (TIF)

S3 Fig Venn diagrams illustrated the relationship between the bacteria communities from different layer. The OTUs was determined at 97% sequences similarity. SUR: surface layer, MID: middle layer, BOT: bottom layer.
(TIF)

S4 Fig **The Weighted Unifrac analysis (A) and PCoA analysis (B) showing the relationship between the bacteria communities from three soil layers in Karamay oilfield**.
(TIF)

S5 Fig CCA ordination plots showing the distribution of bacteria communities response to the soil environmental parameters. Correlation between environmental variables is represented by the length and angle of arrows.
(TIF)
