# Supplementary figures and images for: Vertical stratification of bacteria and the chemical compounds in crude oil-contaminated soil layers of the semi-deserted Dzungharian Basin
Source: PLoS One. 2018 Sep 25;13(9):e0203919. doi: 10.1371/journal.pone.0203919 (PMC6155523; doi:10.1371/journal.pone.0203919)

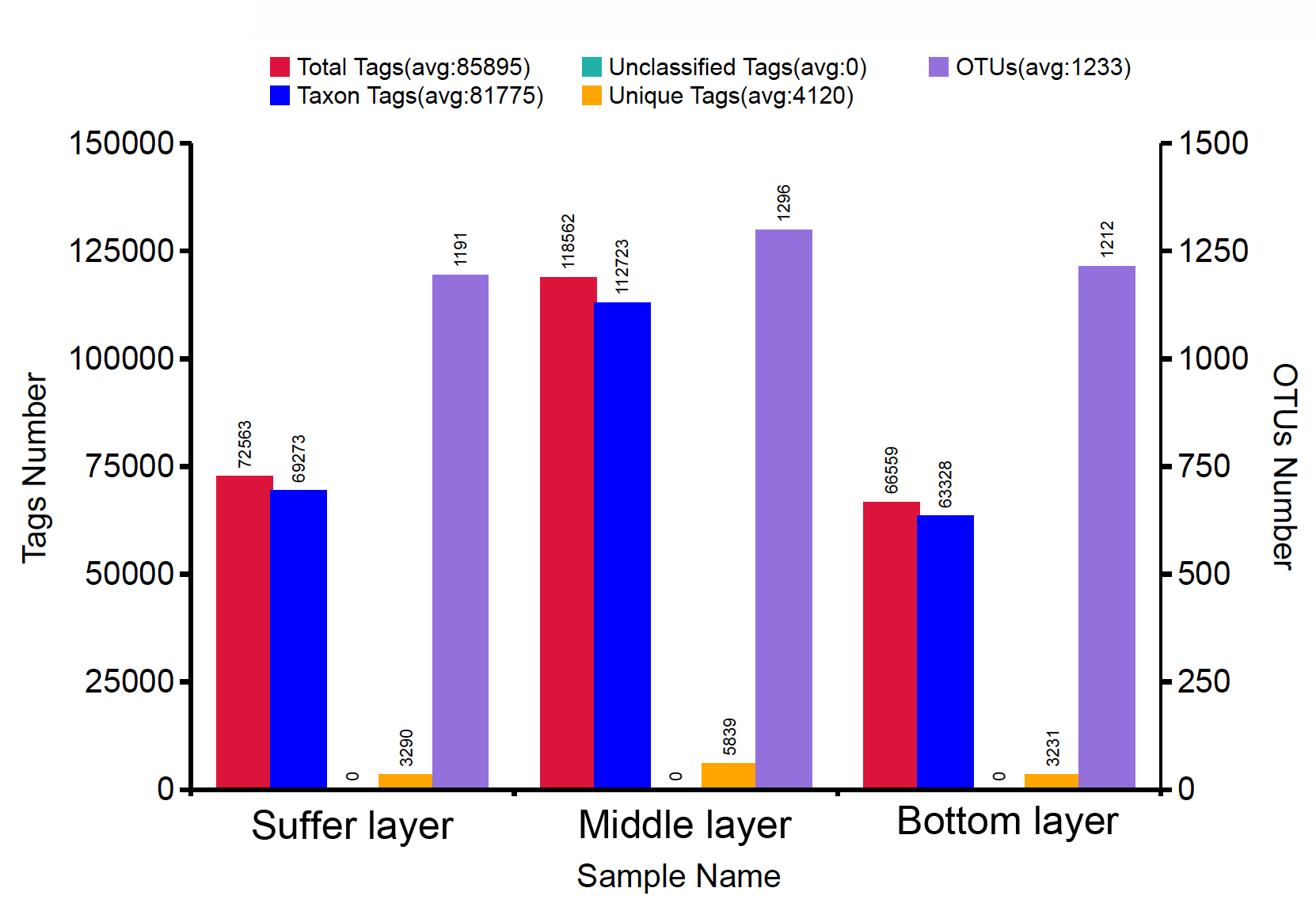

Supplement: S1 Fig — The number of OTUs defined at the level of 97% similarity. (TIF) [file pone.0203919.s001.tif]

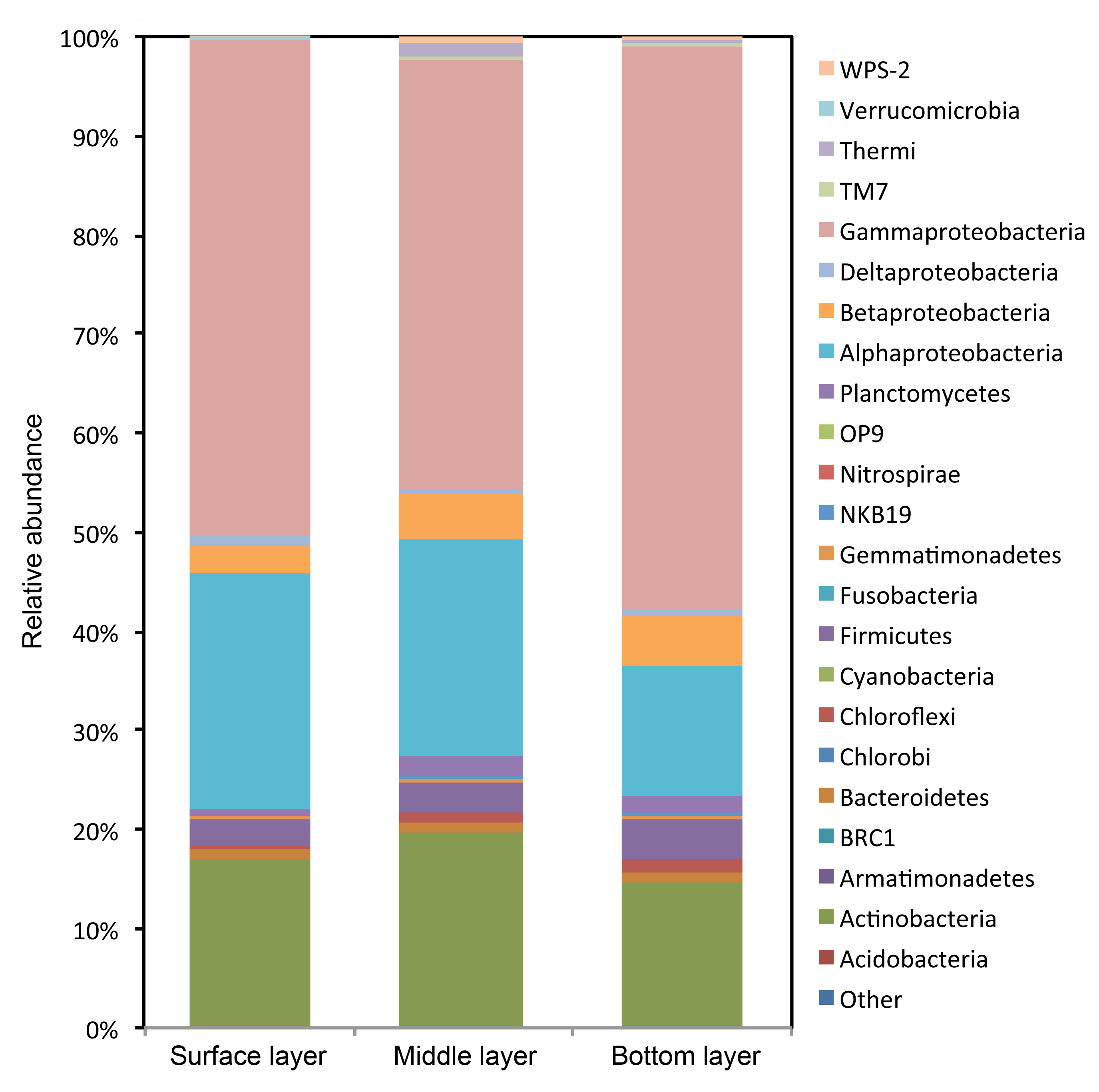

Supplement: S2 Fig — (TIF) [file pone.0203919.s002.tif]

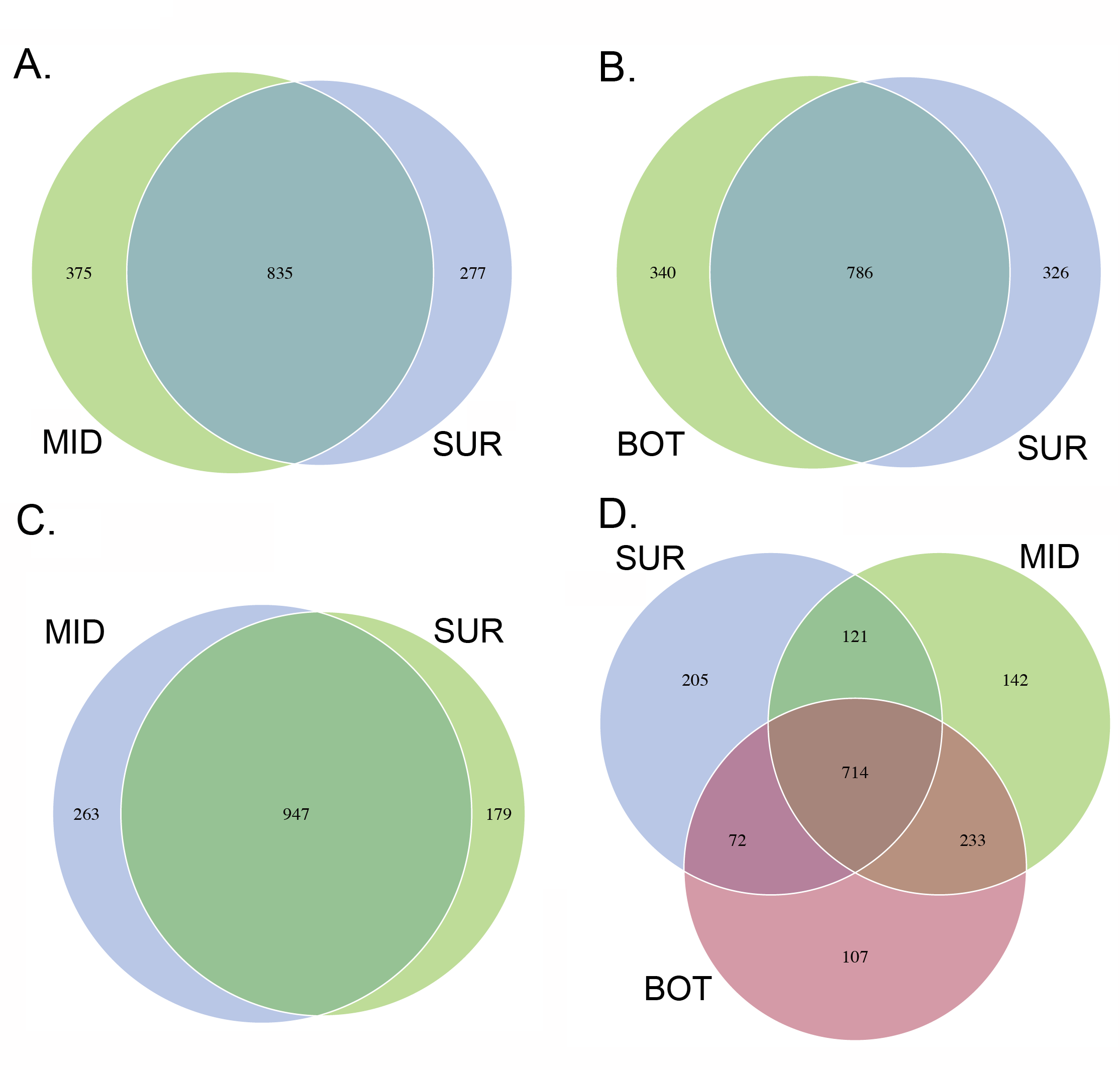

Supplement: S3 Fig — The OTUs was determined at 97% sequences similarity. SUR: surface layer, MID: middle layer, BOT: bottom layer. (TIF) [file pone.0203919.s003.tif]

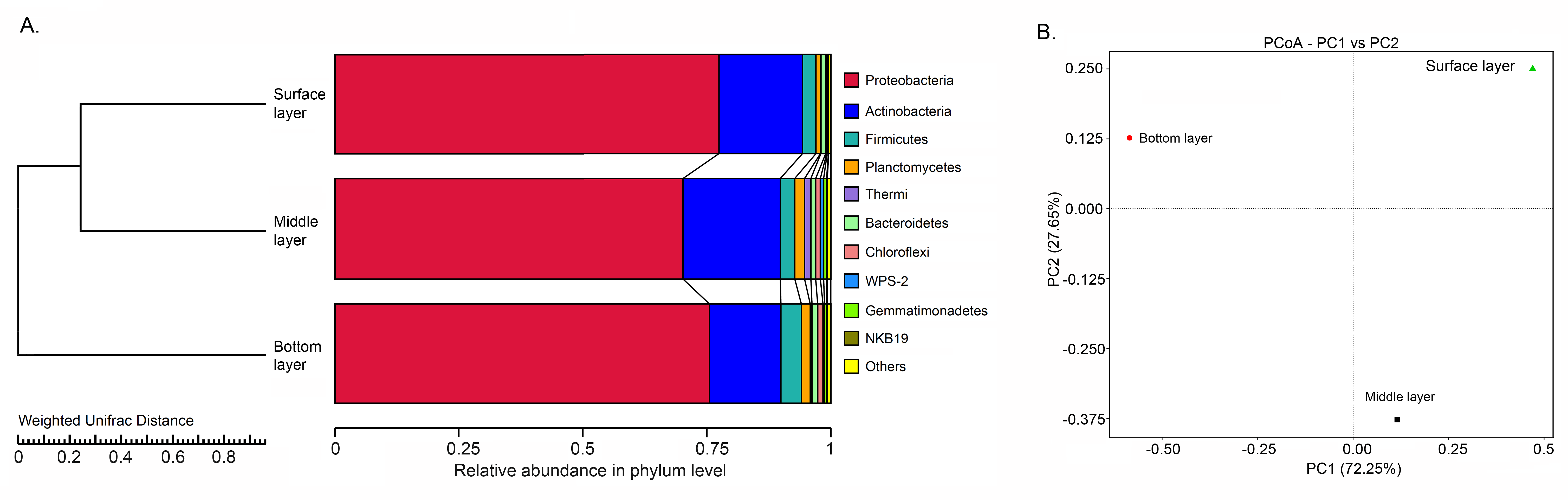

Supplement: S4 Fig — The Weighted Unifrac analysis (A) and PCoA analysis (B) showing the relationship between the bacteria communities from three soil layers in Karamay oilfield. (TIF) [file pone.0203919.s004.tif]

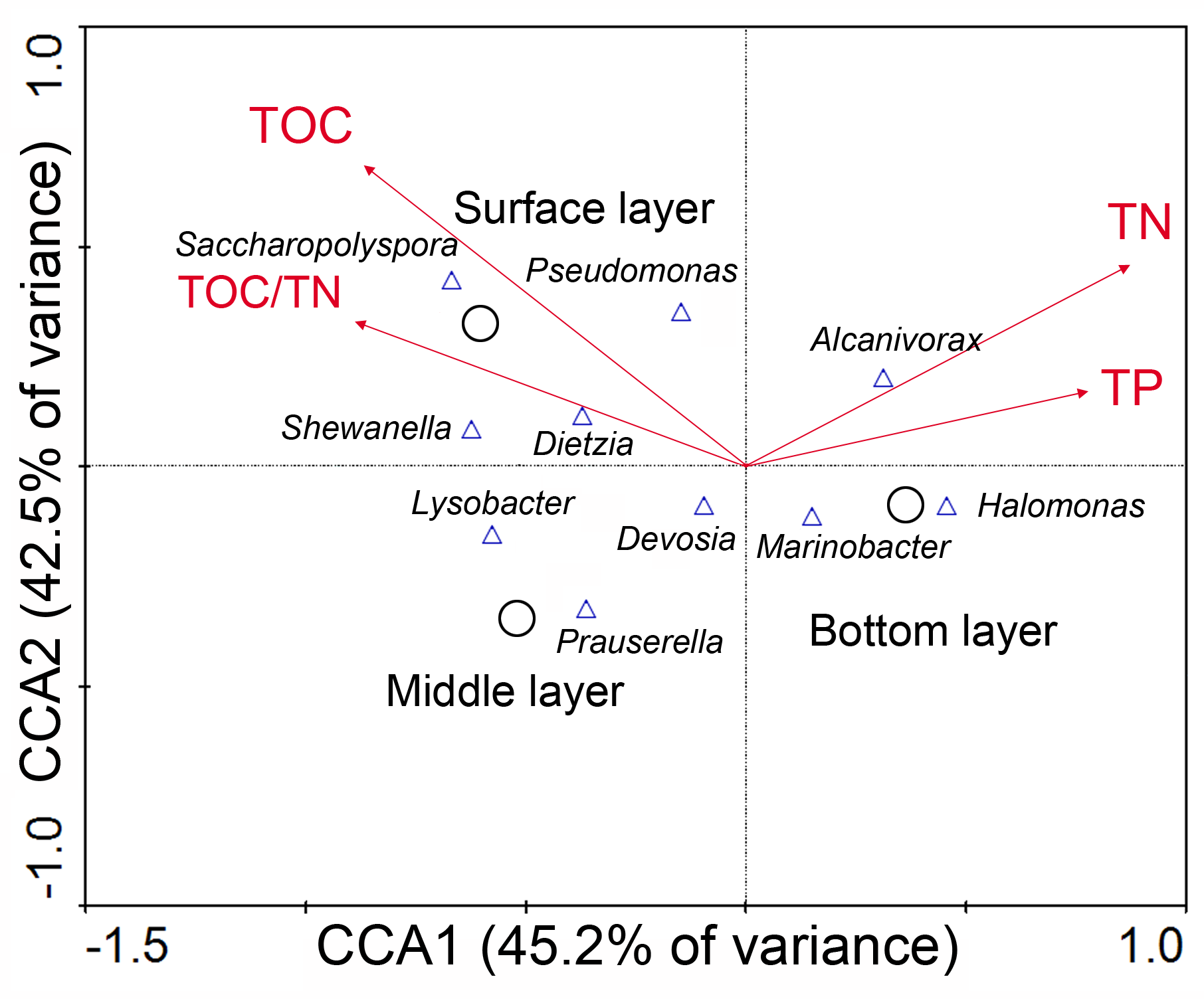

Supplement: S5 Fig — Correlation between environmental variables is represented by the length and angle of arrows. (TIF) [file pone.0203919.s005.tif]
